# Double power-law viscoelastic relaxation of living cells encodes motility trends

**DOI:** 10.1038/s41598-020-61631-w

**Published:** 2020-03-16

**Authors:** J. S. de Sousa, R. S. Freire, F. D. Sousa, M. Radmacher, A. F. B. Silva, M. V. Ramos, A. C. O. Monteiro-Moreira, F. P. Mesquita, M. E. A. Moraes, R. C. Montenegro, C. L. N. Oliveira

**Affiliations:** 1 Departamento de Física, Universidade Federal do Ceará, 60455-970 Fortaleza, Ceará Brazil; 2 Central Analítica, Universidade Federal do Ceará, 60455-970 Fortaleza, Ceará Brazil; 3 Institute of Biophysics, University of Bremen, Otto-Hahn Allee 1, 28359 Bremen, Germany; 4 Departamento de Bioquímica e Biologia Molecular, Universidade Federal do Ceará, 60440-554 Fortaleza, Ceará Brazil; 5 Centro de Biologia Experimental, Universidade de Fortaleza, 60811-905 Fortaleza, Ceará Brazil; 6 Núcleo de Pesquisa e Desenvolvimento de Medicamentos, Universidade Federal do Ceará, 60430-275 Fortaleza, Ceará Brazil

**Keywords:** Collective cell migration, Biomaterials - cells, Biological physics

## Abstract

Living cells are constantly exchanging momentum with their surroundings. So far, there is no consensus regarding how cells respond to such external stimuli, although it reveals much about their internal structures, motility as well as the emergence of disorders. Here, we report that twelve cell lines, ranging from healthy fibroblasts to cancer cells, hold a ubiquitous double power-law viscoelastic relaxation compatible with the fractional Kelvin-Voigt viscoelastic model. Atomic Force Microscopy measurements in time domain were employed to determine the mechanical parameters, namely, the fast and slow relaxation exponents, the crossover timescale between power law regimes, and the cell stiffness. These cell-dependent quantities show strong correlation with their collective migration and invasiveness properties. Beyond that, the crossover timescale sets the fastest timescale for cells to perform their biological functions.

## Introduction

Cell physics has become a very active field of science. In fact, since the end of last century, it has been suggested that physics could deliver biology into another revolution^1^. Since then, the cell turned out to be a breeding ground for interdisciplinary research in order to describe from the point of view of physics its biological functions in both normal and abnormal conditions^2^. Indeed, it is now quite accepted that viscoelastic properties of cells are deeply connected to their multiplication, motility and regulation of disease states^3–10^. For example, many works in the literature have shown that cancer cells are softer and less viscous than normal cells^6,7^. But recently, it was shown that cancer cells exhibit enhanced stiffness when cultured in soft gels as compared to normal cells^9,10^. The reasons for those controversial results are still unclear, but the answers certainly depends on the complicated interplay between the ability of the cells to sense the extracellular matrix rigidity and the consequent reorganization of the cytoskeleton.

Unlike simple viscoelastic materials, cells do not hold characteristic relaxation time, although, most of the studies in the past fit their experimental data with exponential decay^6,11–13^. Instead, the complex architecture of living cells present exquisite viscoelastic properties, making cells exhibit power-law (PL) relaxation. So far, the most used model to describe this cell mechanical behavior (even under pharmacological interventions) is the so called soft-glassy rheology (SGR) model^14,15^. However, this model alone is neither able to describe other behavior commonly observed in cells (such as force generation, prestress and contractile stiffening) nor provide a microscopic origin of such behavior^16^. There are a few studies proposing that cells exhibit a double PL shear modulus, ∣G^*^(*ω*)∣ = *Aω*^*α*^ + *Bω*^*β*^, with *α* > *β*, where the higher and the lower exponents describe, respectively, the fast and slow dynamic response of the cell^17–21^. In this case, the storage modulus $G{\prime} (\omega )$ is well described by a single PL with low exponent of the order of *β* = 0.2 and the loss modulus *G″*(*ω*) described by two PL regimes, with a lower exponent identical to the exponent of $G{\prime} (\omega )$ and a fixed exponent *α* = 1 for all samples. This *ad hoc* combination of PL responses is known as power-law structural damping model, and it was used as the theoretical basis to describe the dynamic rheology of cells in several works^14,15,22–26^. The slow cell response is attributed to the glassy-like regime of the cytoskeleton dynamics that, differently from inert glassy systems, can be remodeled by biological functions such as migration, cytokinesis and mechanotransduction^27^. The origin of the fast cell response is attributed to the entropic response of the f-actin networks^28–35^. These networks exhibit fast PL relaxation with exponent varying between 0.5 and 0.75. Myosin motors (the main source of internal stress in living cells) are responsible for both contractility and fluidization of the cytoskeleton, and the exponent of 0.5 (0.75) is attributed to transiently (permanently) crosslinked f-actin network^36^.

Up to now, the double PL relaxation in cells was only observed by truly oscillatory experiments. AFM force curves are often considered not suitable to probe the fast response of the cells. Here we show that cells do exhibit double PL relaxation compatible with the fractional Kelvin-Voigt viscoelastic relaxation model, and that it can be accurately measured with simple AFM force curves. The double PL relaxation seems to be a universal response of living cells regardless their health state, and a closer analysis of the relaxation exponents may shed new light on the understanding of how diseases develop and how to fight them.

## Results

### Analysis of experimental force curves

**Figure 1:**
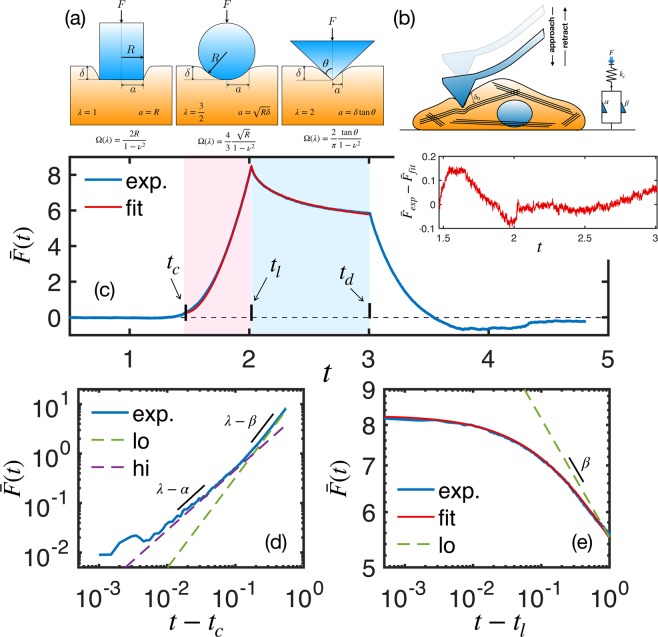
Force curves. (**a**) Contact geometry of common axi-symmetric indenters and their respective force model parameters. (**b**) Schematics of cell indentation and its corresponding fractional Kelvin-Voigt viscoelastic model. (**c**) AFM force curve (blue line) of a AGP01 cell, measured with *f*_*z*_ = 0.25 Hz and *τ*_*d*_ = 1 s, and the double power law fitting (red line) using Eq. (6). The loading and dwell stages are highlighted in blue and pink shaded areas, respectively. The inset graph shows the residual, i.e., the difference between model and experimental force curve during the whole contact region. (**d**) The log-log plot of the loading curve shows the double PL regime where the fast (slow) regime is described by *t*^*λ*−*α*^ (*t*^*λ*−*β*^). A perfectly elastic material, as predicted by Hertz model, would be represented by *t*^*λ*^. (**e**) The log-log plot of the dwell function shows that at very long times after contact the force is described by *t*^−*β*^. $\bar{F}(t)$ is given in units of kPa.

A typical force curve measured with AFM on a single cell is shown by the blue line in Fig. 1, where the loading and dwell stages are highlighted in blue and pink shaded areas, respectively. In the loading part, the piezo expands with constant velocity (controlled by the vertical frequency *f*_*z*_) towards the sample. Before touching the sample surface, the deflection force in the cantilever is zero. When the AFM tip touches the sample at *t* = *t*_*c*_, the force increases until a predetermined maximum force, *F*_*max*_, at *t* = *t*_*l*_. The loading time, *τ*_*l*_ = *t*_*l*_ − *t*_*c*_, is roughly given by $2{f}_{z}\approx {\tau }_{l}^{-1}$. After reaching *F*_*max*_, the piezo stops moving during a time period *τ*_*d*_ (known as dwell time) in which the deflection force relaxes as a consequence of the cell internal relaxation. After the dwell stage, the piezo is retracted with the same speed of the loading stage. The whole force curve can be described by only three input parameters: *f*_*z*_, *F*_*max*_ and *τ*_*d*_, and the data fitting is, in principle, robust enough to work in a wide range of experimental conditions.

### Theoretical model

We derived an analytical force model based on the fractional Kelvin-Voigt viscoelastic relaxation that leads to a double power law $\bar{F}(t)=a{t}^{\lambda -\alpha }+b{t}^{\lambda -\beta }$, shown in Eq. (6), where *a* and *b* depend on the cell type, how fast it is deformed, and on the indenter geometry described by *λ* (see Fig. 1(a)), while *α* and *β* represent the fast and slow relaxation exponents, respectively. Our model fits well the AFM force curve both in the loading and dwell stages, as shown by the red curve in Fig. 1(c). At very short times after contact, the curve is dominated by the fast PL regime, *F*(*t*) ∝ *t*^*λ*−*α*^, and, at some point before achieving *F*_*max*_, the slow PL regime, *F*(*t*) ∝ *t*^*λ*−*β*^, takes over until the end of the dwell stage at very long times after contact, as shown in Fig. 1(d,e). An important issue is whether *τ*_*l*_ is large enough to reveal the crossover between slow and fast response. Although it has been suggested that the transition timescale between fast and slow dynamics, denoted as *t*_*cross*_ (see Methods for mathematical definition), is about 10 ms^17^, this timescale certainly varies from cell to cell. Therefore, it is difficult to anticipate the measuring parameters that shows the double PL only in the loading part. This is why dwell force curves (DFCs) should be adopted rather than conventional force curves, i.e., retract the AFM tip immediately after pushing the surface without giving the cell some time to relax.

**Figure 2:**
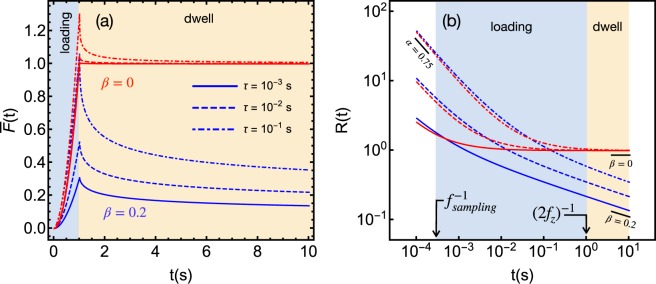
Simulated force curves (**a**) and their corresponding relaxation functions (**b**) calculated with *E*_0_ = 1 kPa, *τ*_*l*_ = 1 s, *τ*_*d*_ = 9 s and *α* = 0.75. $\bar{F}(t)$ and *R*(*t*) are given in units of kPa.

The analysis of simulated force curves of materials with different values of *β* and *τ* (fixed *α* = 0.75), shown in Fig. 2, provides guidelines to observe the double PL regime in DFCs. For a given *τ*, a long dwell relaxation tail is observed for *β* > 0 (*β* < *α*), whereas the amplitude of relaxation is proportional to the ratio *τ*∕*τ*_*l*_. For *β* = 0 the dwell relaxation tail is very short, and becomes nearly absent for very small ratios of *τ*∕*τ*_*l*_. The blue-shaded (yellow-shaded) region represents the time window of the loading (dwell) part of the force curve. The loading part is limited to the left by the shortest measurable time scale (inverse of the AFM sampling frequency ${f}_{sampling}^{-1}$), and to the right by the loading time (${\tau }_{l}\approx {(2{f}_{z})}^{-1}$). The dwell time window is limited to the right by the dwell time. The whole curve comprises nearly five orders of magnitude in time. A successful observation of the double PL relaxation only occurs if the crossing point *t*_*cross*_ lies near the mid point in log scale of the whole measurement window. In principle, it is possible to observe the double PL only in the loading part, but this depends on the ratio *τ*∕*τ*_*l*_. As *t*_*cross*_ is unknown, one can experimentally enlarge the loading time window increasing ${f}_{sampling}^{-1}$ and/or reducing *f*_*z*_. Increasing *τ*_*d*_ enlarges the dwell time window in the region of the low exponent relaxation. In the specific case of *β* = 0 and *τ*∕*τ*_*l*_ = 0.001, the high exponent contribution of *R*(*t*) relaxes very quickly in the first few ms after the onset of the loading curve, such that the system relaxes according the low exponent for most of the loading time. This is a case where the fitting of the low exponent contribution in the loading time will be sufficient, while the fitting of the high exponent may be inaccurate. The opposite case is observed for *τ*∕*τ*_*l*_ = 0.1. The fitting of the high exponent will be accurate, but the fitting of the low exponent may be inaccurate without the dwell part. The same analysis also applies to the cases where *β* > 0. To summarize, the rule of thumb to observe the double PL regime is that the slower the loading stage (smaller *f*_*z*_) the better are the chances to observe it. Combining small *f*_*z*_ with long *τ*_*d*_ (of the order of few seconds), the whole DFC will be long enough such that the slow response (small exponent) will dominate the relaxation in the dwell stage, despite of its appearance in the loading stage. All cells probed in this work clearly reveal the double PL regime in the loading curve for *f*_*z*_ up to 1 Hz.

### Cell mechanical parameters

**Figure 3:**
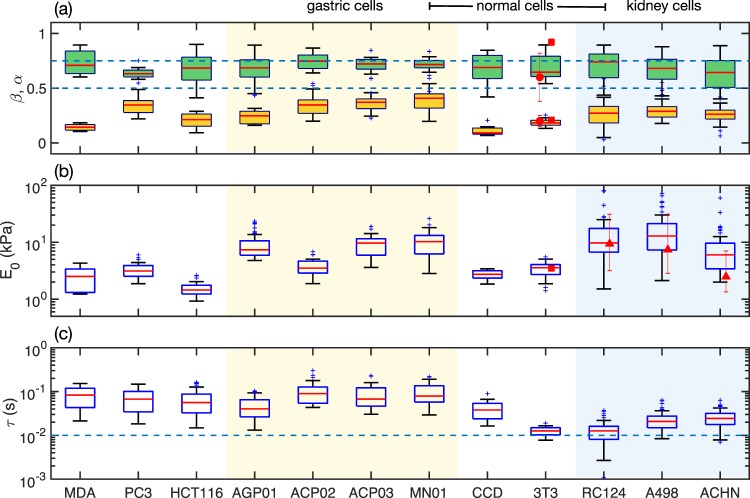
Cell mechanical parameters. Box-and-whisker plots of the fitted parameters. (**a**) Relaxation exponents *α* (fast, green boxes) and *β* (slow, yelow boxes). Horizontal dashed lines represent the lower and upper limits expected for *α*. The red circles and squares represent the values of *α* and *β* measured in previous works using oscillatory methods^20,21^. (**b**) Cell stiffness, *E*_0_. Red triangles and squares represent the values of *E*_0_ measured previously^7,21^. (**c**) Intrinsic timescale, *τ*. The horizontal dashed line represents the value of $\tau ={\omega }_{cross}^{-1}$ measured for bovine airway smooth muscle cells^17^.

 Figure 3 shows the mechanical parameters measured for twelve types of cells, including healthy and cancerous cells from different organs (see descriptions in Methods). The median values of *α*, shown in Fig. 3(a), vary from 0.63 (for PC3 and 3T3) to 0.74 (for ACP02, ACP03, MN01 and RC124). These values are between 0.5 and 0.75 (represented by dashed lines) describing the range of the high frequency response of f-actin networks reported by many groups^28–36^. The small variation of the mean values of *α* among different cell lines is compatible with the cell response attributed to the entropic dynamics of the crosslinked actin filaments in the cytoskeleton subjected or not to prestress^17,36^. On the other hand, the mean value of *β* varies from 0.09 (CCD cell line) to 0.41 (MN01 cell line). This large variation of *β* is explained by the fact that cells are very different in their origin, biological functions and cytoskeleton morphology (Fig. S7 in the Supplementary Material). In general, the dispersion of *α* is larger than of *β* (for the same cell line) because the fast response is only appreciable within few tens of milliseconds after contact. Thus the number of data points in this region is smaller than that of the slow response. This can be improved by increasing the sampling rate of the AFM controller at the expense of higher data storage. However, we did not observe significant difference in the averaged values of both exponents with sampling rates from 2 kHz to 50 kHz (Fig. S4 in the Supplementary Material). Another plausible explanation for the dispersion of *α* and *β* is that the force curves may have been measured in different parts of the cells, with different local cytoskeleton composition.

In order to compare our method with truly oscillatory measurements, we review data from previous studies of 3T3 cell lines (mouse embryonic fibroblasts). The dynamical response of this type of cell was studied combining the AFM with magnetic force modulation within the range 0.1 Hz and 1 kHz^20^. The results exhibited a low response exponent of *β* = 0.2 and fast response exponent of *α* = 0.6 (red circles in Fig. 3(a)). When we analyze the force curves measured in that work with the current model we find *β* = 0.18 and *α* = 0.64. More recently, 3T3 cell lines were also studied in a much wider range of frequencies (from 10^1^ Hz to 10^5^ Hz), where the exponents were found to be *β* = 0.21 and *α* = 0.92 (red squares in Fig. 3(a))^21^. We also applied the current model to the original data of a previous study of the viscoelasticity of kidney cancer cells^7^ (RC124, A498, and ACHN cell lines). The values of *E*_0_ measured here agree well with the values found in that work (red triangles in Fig. 3(b)). The good agreement of our measurements with data reported previously, and obtained with dynamical methods by different research groups and different cells, clearly reveal the potential of our method to study both fast and slow mechanical processes in living cells from simple relaxation data.

### Universal double PL relaxation

To the best of our knowledge, the diversity of cells measured here is larger than most of previous studies. Moreover, the presence of a double PL relaxation in all these samples are ubiquitous. The values of *β* in our measurements are in very good agreement with the values predicted by the soft-glassy rheology model^14^. In the specific case of 3T3 cells, the values of *α* and *β* are in excellent agreement with those found with oscillatory measurements^20,21^. As for the fast response exponent *α*, the measured values lie within the limits established by transiently (*α* = 0.5) and permanently (*α* = 0.75) crosslinked f-actin networks^36^. Such a general behavior makes us conclude that the double PL relaxation is a universal characteristic and that the fractional KV model is an adequate theoretical framework to describe the viscoelastic relaxation of living cells. The transition time between fast and slow dynamics (*t*_*cross*_) varied from cell to cell, but they all ranged from 10 ms to 100 ms (see Fig. S5), in good agreement with the crossover estimated with oscillatory measurements^17^.

Due to the characteristic force ramp of AFM force curves, the high frequency response is probed at small indentations (typically of the order of few hundreds of nanometers and equivalent to the thickness of the cell cortex), and the low frequency response is probed at large indentations (deep in the cell body). In magnetic twisting cytometry experiments, cells are probed either in the surface or in the intracellular regions, depending where the magnetic beads are located, while in oscillatory AFM-based measurements (frequencies up to 1 kHz), a small amplitude sinusoidal signal is superimposed on an initial indentation depth larger (ranging between 1.0 *μm* and 1.5 *μm*) than the thickness of the cell cortex^37^. Concerning whether internal (cytosol) and external (membrane and cortex regions) measurements lead to different responses, it has been shown that the slow exponents, *β*, change slightly when probed inside or outside cells, while the fast exponents, *α*, are region-insensitive^18,19^. It is clear that our method cannot fully decouple the internal and external responses as the AFM tip indents the cells, but the values of *β* measured here span the full range of *β* measured inside and outside cells^19^. However, it is not clear whether the increasing indentation during the force curve measurement induces some prestress in the cell that decreases the exponent *α* of the cytoskeleton network. Besides, as in internalised particle-based rheology experiments^19^, there is always the possibility that the probe (the AFM tip in our case) touches some organelle during the experiment. In the light of the findings of Hoffman *et al*.^19^, we suspect that the slow exponent *β* slightly increases when an organelle is pressed during force curve. In the oscillatory measurements that obtained *α* ≈ 1, the probes were in simultaneous contacts with cells and fluid, thereby subjected to the frequency-dependent response of both. In our measurements, the cantilever is also in contact with the fluid, but moving with speed far below the probes (and only during the loading curve) in the oscillatory experiments, thereby subjected to very small hydrodynamic forces. Besides, our force model is determined from the indentation speed which is a directly measurable quantity, eliminating the need to determine the hydrodynamic drag on the cantilever with approximate models that may not fully account for its contribution^21,23^.

## Discussion

Most of the models used to determine the viscoelastic properties of cells are too simplistic and do not take into account neither their internal organization nor how they respond to the external micro environment. Cells are sophisticated heterogeneous systems composed of a network of biopolymers and organelles immersed in the cytoplasmic fluid, thereby difficult to be mechanically modeled in full detail, whether spatially or in the time. However, our model allows the mechanical description of the cells by a simple set of parameters (*E*_0_, *α*, *β*, and *τ*) that provides not only the mechanics of whole cells but also insights to understand internal biological processes. It is well known from critical phenomena literature that PL behavior can arise from complex structural networks, and that the exponents carry information on the geometry and connectivity of those networks. Bringing these concepts to cell physics, the relaxation exponents of the cells reflect the internal organization of the cytoskeleton. Indeed, confocal images (Fig. S7) reveal different structural organization of the f-actin network among cells with distinct mechanical properties. It is also known that the exponents *α* and *β* are sensitive to pharmacological interventions (and health disorders) that provokes cytoskeleton relaxation, contraction and disruption^15,17,18,21,24^.

Drugs that disrupt the cytoskeleton (e.g. cytochalasin B and D, and latrunculin A and B) are known to reduce dramatically cell stiffness with increasing concentration of the drug^38–40^. However, little is known about their effects on the viscoelastic relaxation of cells. In the few studies addressing this point, Fabry *et al*. shown that cytochalasin D increases the viscous contribution of the cytoplasm, manifested through an increase of the slow relaxation exponent *β* from 0.2 to 0.32 in HASM (human airway smooth muscle) cells^15^. They also tested other four types of cells and a similar increase of *β*. As for latrunculin A, Rigato *et al*. shown that 3T3 cells becomes more viscous at high frequencies, with the fast exponent *α* increasing from 0.92 ± 0.03 to 0.94 ± 0.18, while the slow exponent *β* is nearly unaffected by this drug (reduction from 0.21 ± 0.01 to 0.20 ± 0.01). The inhibition of f-actin crosslinking with CK666 in 3T3 cells increased the elastic character at both low (reduction of *β* from 0.21 to 0.09) and high frequency (reduction of *α* from 0.92 to 0.59) regimes^21^. In addition, it was demonstrated that cytoskeleton relaxation with dibutyryl cAMP reduced increased both fast and slow exponents in human and bovine ASM cells^15,17^, while cytoskeleton contraction with histamine, KCl and calyculin-A reduces both relaxation exponents^15,17,21^.

During physiological processes, the cytoskeleton is constantly remodeled. Such reorganization, essential for cell motility, is regulated by Rho family of small GTPases (mainly Rho, Rac and Cdc42) which are over expressed in many types of cancer cells^41,42^. Thus, if the crosslinking pattern in the cytoskeleton is altered by some disease, we expect modifications in the associated mechanical parameters. Mizuno *et al*. shown that molecular motors (Myosin II and crosslinkers) induce stresses that dramatically affect the stiffness an viscoelastic response of active cytoskeletal networks^43^. Thereby, we speculate that the relaxation exponents possibly carry insights about the kinetics of molecular motors in the cell. As the fast cell response is associated to entropic fluctuations of the cytoskeleton filaments, and the slow response is associated to physiological functions of the cell (e.g. crawling, migration, contraction, invasion), one can regard the crossover timescale between regimes *t*_*cross*_ as how fast cells can perform such functions, and this timescale can be directly compared with primary biological processes such as migration and invasiveness.

The cells studied in this work can be divided between healthy (MN01, CCD, 3T3, RC124) and cancer cells (HCT116, AGP01, ACP02, ACP03, MDA, PC3, A498, ACHN), but such a classification does not favor a direct comparison among them because they are too different in the function they play in living organisms. There are, however, two sets of cells that can be grouped according to their origin, as follows: Group one, with human gastric cells (MN01, AGP01, ACP02, ACP03)^44^; and Group two, with human renal cells (RC124, A498, ACHN)^7,9,10^.

**Figure 4:**
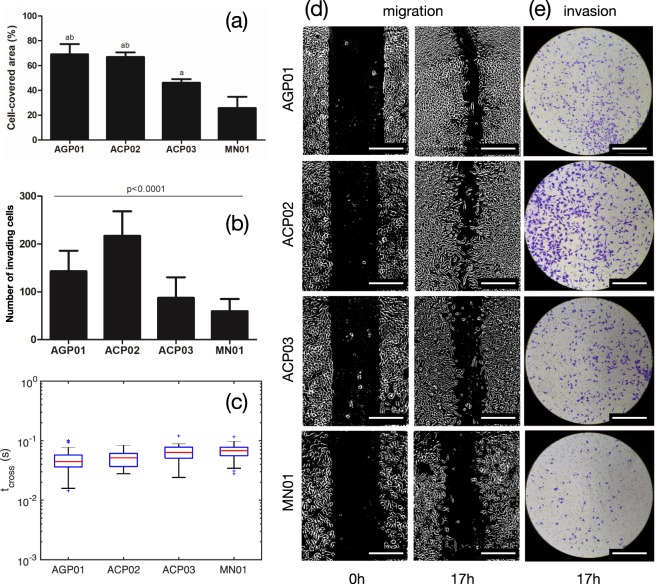
Connection between biological functions and mechanical properties. Comparison of wound healing (WL) (**a**) and invasion (**b**) assays of gastric cell lines. In both cases, data are expressed as the mean ± standard deviation of three independent experiments. In panel (**a**), the letters *a* and *b* represent the statistical comparison with MN01 (p < 0.0001) and with ACP03 (p < 0.001). Significant differences were detected by analysis of variance (ANOVA), followed by Bonferroni’s posttest. (**c**) Box-and-whisker plots of the crossover timescale between fast and slow power-law regimes. (**d**) Images of the gastric cell at an initial time (0 h) and after 17 h culture for stimulating motility. (**e**) Images of inserts with invading gastric cells after 17 h of culture. The scale bar is of 200 *μm*. The choice of 17 h was based on the doubling time which is about 24 h for these cell lines (AGP01, ACP02, ACP03 and MN01), avoiding interference of cell division in the analysis.

In Group one (gastric cells), ACP02 and ACP03 cells are taken from primary non-metastatic adenocarcinoma tumors, and histologically classified diffuse as intestinal types, respectively, while AGP01 cells are taken from the ascitic fluid of a metastatic adenocarcinoma. MN01 are healthy gastric cells. In addition to the mechanical study, we also performed migration (*M*) and invasiveness (*I*) assays. Figure 4(a) shows that the migration abilities of AGP01 and ACP02 are equivalent and enhanced compared to ACP03 and MN01. The migration measurements in this group are summarized as *M*_*AGP*01_ ≈ *M*_*ACP*02_ > *M*_*ACP*03_ > *M*_*MN*01_. The migration of the gastric cells inversely correlates with the timescale between PL regimes *t*_*cross*_, as shown in Fig. 4(c). From the invasiveness viewpoint (Fig. 4(b)), ACP02 is the most invasive cell line in this group, despite of the fact that it is originated from a non-metastatic tumor. The invasiveness of the gastric cells is summarized as *I*_*ACP*02_ > *I*_*AGP*01_ > *I*_*ACP*03_ > *I*_*MN*01_, and this comparative behavior inversely correlates with cell stiffness *E*_0_ (Fig. 3(b)).

The strong correlation between motility functions and the mechanical parameters of the cells of Group one is not accidental. Cell crawling requires an extremely coordinated sequences of de-polymerization and re-polymerization of cytoskeleton filaments in the cytoplasmic crowd. The faster those processes occur, the farther cells can migrate. As for invasion, the underlying processes are more complicated. In the present assay, invasion occur from the top to bottom, and cells first open their way through the extracellular matrix layer using metalloproteinases that degrades the matrix polymers^45–47^. After cells reach the porous membrane, they flow passively in the pores. This process becomes easier for cells with reduced stiffness and viscosity. This is the physical reason why ACP02, the less stiff gastric cell in group 1, exhibits the highest invasiveness. Interestingly, ACP02 originated from a diffuse tumor^44^, exhibiting an infiltrative nature as opposed to focal tumors which are more spatially circumscribed. This is probably caused by the large invasiveness of these cells in adjacent tissues. One must remind that the internal processes involved in cell migration and invasion occur within the time domain of the slow viscoelastic relaxation. A completely different process occur when cells try to invade soft nonporous materials. In this case, cells have to either use a polymerization ratchet process, or use myosins to generate forces. In both cases the cell will become stiffer. This is supported by a recent work that demonstrated that cancer cells on soft gels appear stiffer than normal cells in the same substrate, and they tend to invade the gels while normal cells not^9^.

In Group two (renal cells), A498 and ACHN are carcinoma and metastatic adenocarcinoma, respectively, while RC124 are normal cells. The migration properties of those cells have been recently reported^9^, and it was found that *M*_*RC*124_ > *M*_*A*498_ > *M*_*ACHN*_. As in group 1, the crossover timescales (*t*_*cross*_) between PL regimes are inversely proportional to the migration properties of those cells. Concerning the exponents, the slow exponent is nearly identical in all cells, but the fast exponent of the cancer cells is lower than that of the normal cell. This renders to ACHN (the metastatic cell line) the most solid-like character in this group. In group one, the metastatic AGP01 line also exhibited the most solid-like character (Fig. S5). However, cells from Group two seem to disobey the expected trend, namely, that cells become less viscous as the cancer aggressiveness progresses^6^. In this group, normal (RC124) and metastatic (ACHN) cells exhibit similar apparent viscosities (See Fig. S5 in the Supplementary Material).

### Concluding remarks

We have measured the viscoelastic relaxation of livings cells in time domain with simple AFM force curves. Our result show that the fractional Kelvin-Voigt model provides a reliable theoretical framework to describe cell mechanics. All cells measured in this work unequivocally exhibit double power-law relaxation, and the crossover timescale between power-law regimes is intrinsically connected to the collective migration of the cells. Concomitantly, the collective invasiveness rate of cells is associated to lower cell stiffness.

## Methods

### Cell lines

The cell lines studied in this work are: human colon cancer cells (HCT116 line); human gastric normal (MN01) and cancer cells (AGP01, ACP02, ACP03); human breast cancer (MDA-MB-231); human prostate cancer cells (PC3); human normal skin fibroblasts (CCD); mouse embryonic fibroblasts (3T3); and human kidney normal (RC124) and cancer cells (A498, ACHN). The AFM data of 3T3, RC124, A498, and ACHN lines were taken from previous works, where the measured force curves were re-analyzed with the present method to determine their mechanical parameters^7,20^.

### Cell culture

Cells were grown in high-glucose Dulbecco’s Modified Eagle’s Medium (GIBCO, USA) supplemented with 10% fetal bovine serum (GIBCO, USA) and 1% penicillin-streptomycin, and incubated at 37 °C in 5% CO_2_. Before AFM measurements, one third of medium was replaced by PBS solution to keep pH stable out of the incubator. All measurements were obtained up to 2 hours after cells were moved out of the incubator.

### Migration (wound healing) assay

Cells were seeded in twelve well-plate and cultured until reaching 80–90% confluence. A cell-free area is created through mechanical damage (scratch) in the center of the well. The scratches were made using a sterile pipette tip in the middle of well to create the gap. The well-plates were photographed after scratch (0 h) and at the final culture time (17 h). This time was chosen based on the doubling time for the cell lines (AGP01, ACP02, ACP03 and MN01) avoiding interference of cell division in the analysis. Images were analyzed to count cells in the gap using ImageJ. Data were expressed as the mean ± standard deviation of three independent experiments and groups were statistically compared by analysis of variance (ANOVA) followed by Bonferroni’s posttest.

### Invasion assay

Membranes with 8 *μ*m pores were inserted in six-well plated, then coated with Matrigel (BD Biosciences, San Jose, CA, USA) and stored at 37 °C for solidification. The lower chamber of the plate was filled with medium containing 10% of serum to stimulate chemo-attraction of cells. The upper chamber was filled with serum-free medium at a density of 15 × 10^4^ cells per insert. After 17 h of re-culture, all cells and Matrigel on the top of inserts were removed, and cells that invaded the matrigel and porous membrane to the lower side of inserts were fixed with absolute methanol and stained with crystal violet 0.6%. The cells in the lower side inserts were counted using ImageJ software. Thirty microscopic fields were counted per insert at a magnification of 400×. Data were expressed as the mean ± standard deviation of three independent experiments and groups were statistically compared by analysis of variance (ANOVA) followed by Bonferroni’s posttest.

### Confocal imaging

Cells were fixed with paraformaldehyde (4% in PBS for 15 min), permeabilized with Triton X-100 (0.5% in PBS for 30 min), and treated with BSA (3% in PBS for 60 min) for blocking at room temperature (25 °C). F-actin filaments were stained with phalloidin (5 *μ*g/ml in PBS). Nuclei were stained 4${\prime}$,6-diamidine-2-phenylindole hydrochloride (DAPI) (100 ng/ml in PBS). Confocal images were obtained at room temperature (25 °C) with a laser scanning confocal microscopy system LSM 710 (Zeiss, Jena, Germany) with excitation at 488 nm and emission between 515 nm and 540 nm.

### AFM measurements

The dwell force curves were then measured in an Asylum MFP3D-BIO coupled to an inverted optical microscope Nikon IX51. We used AFM cantilevers of nominal spring constant of 0.02 N/m with pyramidal tip (with nominal height of 3.5 *μm*). The cantilevers were calibrated with thermal method prior to measurements and the actual spring constants differed from the nominal value by less than 10%. In order to probe deeper parts of the cytoskeleton of cells, the maximum force *F*_*max*_ applied to the cells ranged between 4 nN and 10 nN, depending on the cell stiffness. The maximum indentation depths ranged between 1 *μm* and 3 *μm*. We adopted a ramp size of 3 *μm*. The indentation speed is controlled by tuning the vertical frequency *f*_*z*_ (0.25 Hz–4.0 Hz) during cantilever approach, resulting in cantilever speeds between 1.5 *μm*∕*s* and 24 *μm*∕*s*, and *τ*_*l*_ between 100 ms and 2 s. Dwell times *τ*_*d*_ between 1 s–5 s were used. The force measurements were performed at room temperature (25 °C) in nearly identical conditions. All AFM experiments were performed with 2 h to minimize the role of evaporation that may cause osmotic pressures in the cells.

### Force curve modeling

The characterization of viscoelastic properties through indentation tests involves the application of a controlled force *F*(*t*) followed by the measurement of the resulting indentation depth *δ*(*t*). In time domain, the force-indentation relationship is given by^48–50^

1 $$F(t)=\Omega (\lambda ){\int }_{0}^{t}R(t-t{\prime} )\frac{d{\delta }^{\lambda }(t{\prime} )}{dt{\prime} }dt{\prime} ,$$

where *λ* and Ω(*λ*) are parameters related to the indenter geometry (Fig. 1(a,b)) and *R*(*t*) is the time-dependent relaxation function that describes the material properties. *R*(*t*) is connected to the dynamical shear modulus by ${G}^{\ast }(\omega )=i\omega {\int }_{0}^{\infty }R(t)\exp (-i\omega t)dt$. In AFM force curves *F*(*t*) and *δ*(*t*) are directly measured while *R*(*t*) is unknown. In general, some viscoelastic model for *R*(*t*) is adopted to explain the measured data with the help of the hints provided by *F*(*t*).

### Viscoelastic relaxation

PL relaxation is described by the framework of fractional calculus through the definition of a fractional element whose constitutive stress-strain equation is defined as *σ*(*t*) ∝ *d*^*n*^*ϵ*(*t*)∕*dt*^*n*^, where *d*^*n*^∕*dt*^*n*^ is a fractional derivative for 0 ≤ *n* ≤ 1. In the limit *n* = 0 (*n* = 1) the sample behaves as purely solid (liquid)^51–54^. As cells exhibit two PL regimes, the generalized version of the well known Kelvin-Voigt (KV) model describes very well the viscoelastic properties of living cells. The relaxation function of fractional KV model is given by (see Section 1 of the Supplementary Material)

2 $$R(t)=\frac{{E}_{0}}{\Gamma (1-\alpha )}{\left(\frac{t}{\tau }\right)}^{-\alpha }+\frac{{E}_{0}}{\Gamma (1-\beta )}{\left(\frac{t}{\tau }\right)}^{-\beta },$$

where *E*_0_ is the cell stiffness, *τ* is a cell-dependent intrinsic timescale that roughly describes the ratio between effective viscosity and stiffness, and *α* and *β* are the fast and slow response exponents, respectively. The complete gamma function Γ(*x*) is an extension of the factorial to complex and real number arguments. It is related to the factorial by Γ(*n*) = (*n* − 1)!. The crossing time between regimes is given by

3 $${t}_{cross}=\tau \,{\left[\frac{\Gamma (1-\beta )}{\Gamma (1-\alpha )}\right]}^{\frac{1}{\alpha -\beta }}.$$

In the frequency domain, the corresponding shear modulus is given by G^*^(*ω*) = *E*_0_ (*iωτ*)^*α*^ + *E*_0_ (*iωτ*)^*β*^, and the crossover frequency between regimes is *ω*_*cross*_ = *τ*^−1^. The dynamic viscosity *η*(*ω*) and phase angle *θ*(*ω*) at *ω*_*cross*_ = *τ*^−1^ are respectively given by

4 $$\eta ({\tau }^{-1})=2{E}_{0}\tau \,\sin \,\left[\frac{(\alpha +\beta )\pi }{4}\right]\,\cos \,\left[\frac{(\alpha -\beta )\pi }{4}\right],$$

5 $$\theta ({\tau }^{-1})=\frac{(\alpha +\beta )\pi }{4}.$$

Within the framework of the fractional Kelvin-Voigt (KV) viscoelastic model^51^, the exact form of the force curves in the loading and dwell stages are, respectively, (derivation in Sections 2 and 3 of the Supplementary Material)

6 $$\begin{array}{lll}{\bar{F}}_{l}(t) & = & {E}_{0}\Gamma (\lambda +1)\,{\left(\frac{t}{{\tau }_{l}}\right)}^{\lambda }\,\left[\frac{1}{\Gamma (\lambda +1-\alpha )}{\left(\frac{t}{\tau }\right)}^{-\alpha }+\frac{1}{\Gamma (\lambda +1-\beta )}{\left(\frac{t}{\tau }\right)}^{-\beta }\right]\,\,\,{\rm{for}}\,\,\,t\le {\tau }_{l},\\ {\bar{F}}_{d}(t) & = & {E}_{0}\Gamma (\lambda +1)\,{\left(\frac{t}{{\tau }_{l}}\right)}^{\lambda }\,\left[\frac{I({\tau }_{l}/t;\lambda ,1-\alpha )}{\Gamma (\lambda +1-\alpha )}{\left(\frac{t}{\tau }\right)}^{-\alpha }+\frac{I({\tau }_{l}/t;\lambda ,1-\beta )}{\Gamma (\lambda +1-\beta )}{\left(\frac{t}{\tau }\right)}^{-\beta }\right]\,\,\,{\rm{for}}\,\,\,{\tau }_{l}\le t\le {\tau }_{l}+{\tau }_{d},\end{array}$$

where *t* is measured from the contact point. *I*(*x*; *a*, *b*) is the regularized incomplete beta function. The force curves are normalized by ${F}_{0}=\Omega (\lambda ){\delta }_{0}^{\lambda }$, where *δ*_0_ is the maximum indentation depth achieved in the loading stage. In the limit of a single fractional element with its corresponding relaxation exponent being equals to zero, the loading force model describes the well known Hertz model for purely elastic materials^55^.

### Data analysis

We measured approximately 10 cells for each cell line, near the highest point of each cell. For each cell, we acquired 5 force curves with *f*_*z*_ = 0.25 Hz, 0.5 Hz, 1.0 Hz, 2.0 Hz and 4.0 Hz (alternating low and high frequencies and with 30 seconds between measurements) to improve the statistics of the fast relaxation exponent that tends to dominate the approach curve for higher *f*_*z*_. The fitted parameters of approximately 50 force curves were used to construct the box-and-whisker plots of Fig. 3. The exceptions are the force curves of 3T3, RC124, A498 and ACHN cells, which were originally measured with *f*_*z*_ = 1.0 Hz. Fortunately, all cells probed in this work revealed the double PL regime in the loading curve for *f*_*z*_ up to 1.0 Hz. Regarding the appearance of the data in Fig. 3 and all other figures containing box plots (Figs. 4, S4 and S5), the central mark indicates the median, and the bottom and top edges of the box indicate the 25th and 75th percentiles, respectively. The whiskers extend to the most extreme data points not considered outliers (plotted individually using the + symbol).

## Supplementary information

Supplementary material.
