# Supplementary material for: Double power-law viscoelastic relaxation of living cells encodes motility trends
Source: Sci Rep. 2020 Mar 16;10:4749. doi: 10.1038/s41598-020-61631-w (PMC7075927; doi:10.1038/s41598-020-61631-w)
Supplement: Supplementary file 1 — Supplementary material. [file 41598_2020_61631_MOESM1_ESM.pdf]

Supplementary Material: Double power-law viscoelastic relaxation of living cells encodes motility trends

J. S. de Sousa[1], R. S. Freire[2], F. D. Sousa[1], M. Radmacher[3], A. F. B. Silva[4], M. V. Ramos[4], A. C. O. Monteiro-Moreira[5], F. P. Mesquita[6], M. E. A. Moraes[6], R. C. Montenegro[6], and C. L. N. Oliveira[1]

[1]Departamento de Física, Universidade Federal do Ceará, Fortaleza, Ceará, Brazil
[2]Central Analítica, Universidade Federal do Ceará, Fortaleza, Ceará, Brazil
[3]Institute of Biophysics, University of Bremen, Bremen, Germany
[4]Departamento de Bioquímica e Biologia Molecular, Universidade Federal do Ceará, Fortaleza, Ceará, Brazil
[5]Centro de Biologia Experimental, Universidade de Fortaleza, Fortaleza, Ceará, Brazil
[6]Núcleo de Pesquisa e Desenvolvimento de Medicamentos, Universidade Federal do Ceará, Fortaleza, Ceará, Brazil

1 Generalized Kelvin-Voigt model

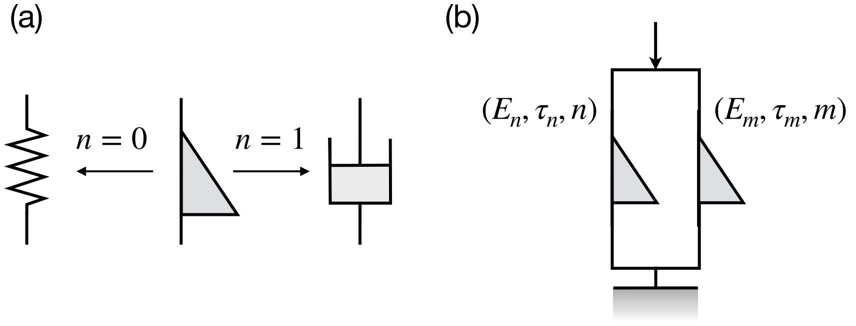

Figure S1: (a) Representation of a single fraction element. (b) The fractional Kelvin-Voigt model.

The constitutive stress-strain equation of a single fractional element, as shown in Figure S1(a), is given by

$$\sigma(t) = \Lambda \frac{d^n \epsilon(t)}{dt^n}, \tag{1}$$

where $\sigma(t)$ is the stress, $\epsilon(t)$ is the strain, d^n/dt^n is the fractional derivative operator ($0 \leq n \leq 1$), and Λ is a material parameter (with SI units of Pa.s^n). One can write $\Lambda = E\tau^n$, such that a single fractional element is described by three parameters (E, τ, n). The fractional element interpolates between two responses: for $n = 0$ for a Hookean elastic spring that can be described only by E (since $n = 0$, τ becomes meaningless) , and $n = 1$ for a Newtonian dashpot. Figure S1(b) shows the fractional KV model, whose stress-strain constitutive equation is given by

$$\sigma(t) = E\tau^n \frac{d^n \epsilon(t)}{dt^n} + E\tau^m \frac{d^m \epsilon(t)}{dt^m}, \tag{2}$$

where $\tau = (E_m \tau_m^m / E_n \tau_n^n)^{1/(m-n)}$ and $E = E_m(\tau_m/\tau)^m$. The Laplace transform of the fractional derivative is given by

$$\mathcal{L}\left[\frac{d^n f(t)}{dt^n}\right] = s^n \tilde{f}(s) - \sum_{k=0}^{m-1} s^{n-k-1} f^{(k)}(0),\ m-1 < n \leq m. \tag{3}$$

Applying the above Laplace transform definition in the fractional constitutive equation (using $\sigma(0) = 0$ and $\epsilon(0) = 0$ as initial conditions), one obtains

$$\tilde{\sigma}(s) = (E_0 \tau^n s^n + E_0 \tau^m s^m)\tilde{\epsilon}(s). \tag{4}$$

Under the hypothesis of causal histories, the stress-strain relationship can be written as

$$\sigma(t) = \int_0^t R(t-t') \frac{d}{dt'} \epsilon(t') dt', \tag{5}$$

whose Laplace transform is $\tilde{\sigma}(s) = s\tilde{R}(s)\tilde{\epsilon}(s)$. Thus, one obtains

$$\tilde{R}(s) = E(\tau^n s^{n-1} + \tau^m s^{m-1}). \tag{6}$$

Applying the inverse Laplace transform, the relaxation function in time domain reads

$$R(t) = \frac{E}{\Gamma(1-m)}\left(\frac{t}{\tau}\right)^{-m} + \frac{E}{\Gamma(1-n)}\left(\frac{t}{\tau}\right)^{-n}. \tag{7}$$

The complex shear modulus is connected to the relaxation function as $G^*(\omega) = i\omega \int R(t) e^{i\omega t} dt$. For the fractional KV model, $G^*(\omega)$ is given by

$$G^*(\omega) = E(i\omega\tau)^m + E(i\omega\tau)^n. \tag{8}$$

2 Force curves determination

The force curves are obtained by solving the integral

$$F(t) = \Omega(\lambda) \int_0^t R(t-t') \frac{d\delta^\lambda(t')}{dt'} dt', \tag{9}$$

where λ and $\Omega(\lambda)$ are parameter related to the indenter geometry (see Table 1), and $R(t)$ is the time-dependent relaxation function. Thus, for a dwell force curve, the load (l), dwell (d) and unload (u) curves are, respectively, given by

$$\bar{F}_l(t) = \int_0^t R(t-t') \frac{d\bar{\delta}_a^\lambda(t')}{dt'} dt' \quad (t \leq \tau_l), \tag{10}$$

$$\bar{F}_d(t) = \int_0^{\tau_l} R(t-t') \frac{d\bar{\delta}_a^\lambda(t')}{dt'} dt' + \int_{\tau_l}^t R(t-t') \frac{d\bar{\delta}_d^\lambda(t')}{dt'} dt' \quad (\tau_l \leq t \leq \tau_l + \tau_d), \tag{11}$$

$$\bar{F}_u(t) = \int_0^{\tau_l} R(t-t') \frac{d\bar{\delta}_a^\lambda(t')}{dt'} dt' + \int_{\tau_l}^{\tau_l+\tau_d} R(t-t') \frac{d\bar{\delta}_d^\lambda(t')}{dt'} dt' + \int_{\tau_l+\tau_d}^t R(t-t') \frac{d\bar{\delta}_r^\lambda(t')}{dt'} dt' \quad (t \geq \tau_l + \tau_d) \tag{12}$$

Table 1: Dependence of the parameters λ and $\Omega(\lambda)$ on the indenter geometry. Below, ν represents the Poisson ratio and δ is the indentation.

Geometry	λ	$\Omega(\lambda)$	contact radius	Obs.
flat cylinder	1.0	$\frac{2R}{(1-\nu^2)}$	R	R is the indenter radius
spherical	1.5	$\frac{4}{3}\frac{\sqrt{R}}{(1-\nu^2)}$	$\sqrt{R\delta}$	R is the indenter radius
conical	2.0	$\frac{2}{\pi}\frac{\tan\theta}{(1-\nu^2)}$	$\delta\tan\theta$	θ is the half-opening angle

where τ_l is the loading time. The indentation history $\delta(t)$ is then normalized by the maximum indentation δ_0 achieved at $t = \tau_l$, such that $\bar{\delta}(t) = \delta(t)/\delta_0$, and the force at each stage is also normalized by $\bar{F}(t) = F(t)/(\Omega(\lambda)\delta_0^\lambda)$.

The load $\delta_l(t)$ and unload $\delta_u(t)$ indentation profiles are well described by linear functions in time, while the dwell profile $\delta_d(t)$ is represented by a smooth increasing function in time $f(t)$ (see Figure 1 in the main manuscript). More specifically, one can write

$$\delta_l(t) = \delta_0 \frac{t}{\tau_l}, \tag{13}$$

$$\delta_d(t) = \delta_0[1 + f(t - \tau_l)], \tag{14}$$

$$\delta_u(t) = \delta_d(\tau_l + \tau_d) \left[1 - \frac{t - (\tau_l + \tau_d)}{\tau_u}\right]. \tag{15}$$

In the above expressions τ_l,τ_d and τ_u represent the duration of the each stage, δ_0 is the indentation at the end of the loading curve, and $f(t)$ is a smooth function of time, such that one can assume $df/dt \approx 0$. For the sake of completeness, we included the unload curve in the expressions above, but we will disregard this part because all the information we seek can be extracted from both loading and dwell curves. In Figure S2 we show a typical force curve (conventional and dwell) measured in the cells. Clearly, the indentation profile during approach can be well described by a linear function in time, while the indentation in the dwell portion is nearly constant, having increased only 75 nm in the dwell part.

3 Analytical integration

Plugging $R(t)$ of the fractional KV model in Eq. 9, we obtain two types of integrals

$$I_{1a} = \frac{\lambda}{\tau_l^\lambda} \frac{1}{\Gamma(1-n)} \int_0^t t'^{\lambda-1} \left(\frac{t-t'}{\tau}\right)^{-n} dt', \tag{16}$$

$$I_{1b} = \frac{\lambda}{\tau_l^\lambda} \frac{1}{\Gamma(1-n)} \int_0^{\tau_l} t'^{\lambda-1} \left(\frac{t-t'}{\tau}\right)^{-n} dt'. \tag{17}$$

In both integrals, the transformation $u = t'/t$ makes the time dependence of the force functions more evident. We can group I_{1a} and I_{1b} in a single expression that reads

$$I_{1x} = \frac{\lambda}{\Gamma(1-n)} \left(\frac{t}{\tau_l}\right)^\lambda \left(\frac{t}{\tau}\right)^{-n} B(x; \lambda, 1-n), \tag{18}$$

where $x = 1$ for I_{1a}, and $x = \tau_l/t$ for I_{1b}. $B(x; \lambda, 1-n)$ is the incomplete Beta function

$$B(x; a, b) = \int_0^x u^{a-1}(1-u)^{b-1} du, \tag{19}$$

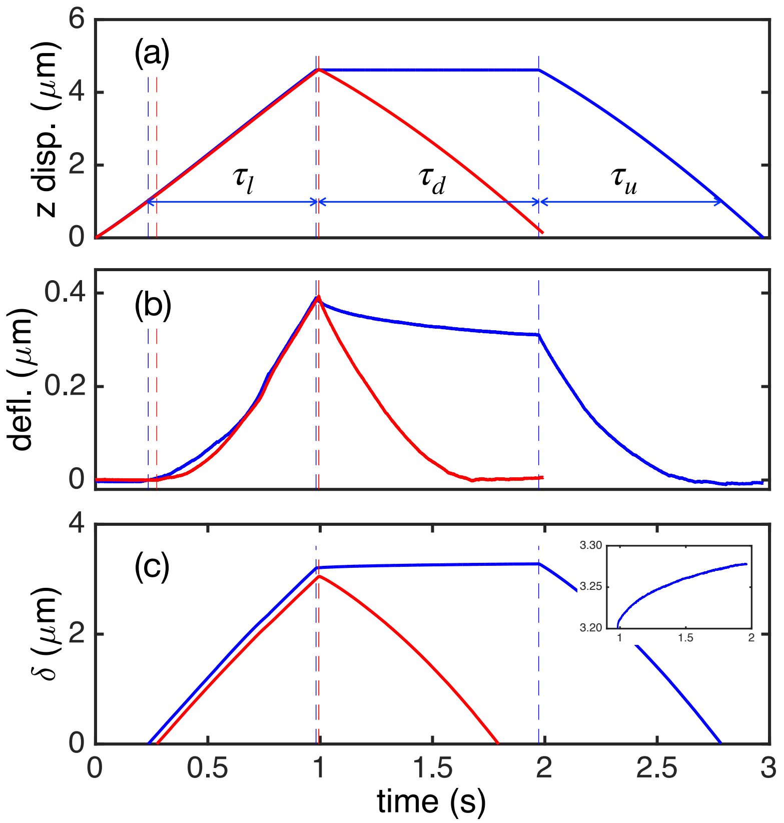

Figure S2: (a) Piezo displacement in conventional (red) and dwell (blue) force curves. (b) Deflection signal measured AGP01 cells with $f_z = 1$ Hz. (c) Indentation depth as function of time. The inset shows that the indentation exhibits small variation in the dwell stage, thereby it can be considered nearly constant.

that obeys $B(1; a, b) = B(a, b)$, and $B(a, b) = \Gamma(a)\Gamma(b)/\Gamma(a + b)$ is the Beta function. Finally, integrals I_{1x} can be cast in the following compact form

$$I_{1x} = \Gamma(\lambda + 1)\left(\frac{t}{\tau_l}\right)^{\lambda} \frac{I(x; \lambda, 1 - n)}{\Gamma(1 + \lambda - n)}\left(\frac{t}{\tau}\right)^{-n}, \tag{20}$$

where $I(x; a, b) = B(x; a, b)/B(a, b)$ is the regularized incomplete Beta function, $x = 1$ for I_{1a}, and $x = \tau_l/t$ for I_{1b}. The final analytical form of the force curves within the fractional KV framework is given by

$$\bar{F}_l(t) = E_0\Gamma(\lambda + 1)\left(\frac{t}{\tau_l}\right)^{\lambda}\left[\frac{1}{\Gamma(\lambda + 1 - \alpha)}\left(\frac{t}{\tau}\right)^{-\alpha} + \frac{1}{\Gamma(\lambda + 1 - \beta)}\left(\frac{t}{\tau}\right)^{-\beta}\right], \tag{21}$$

$$\bar{F}_d(t) = E_0\Gamma(\lambda + 1)\left(\frac{t}{\tau_l}\right)^{\lambda}\left[\frac{I(\tau_l/t; \lambda, 1 - \alpha)}{\Gamma(\lambda + 1 - \alpha)}\left(\frac{t}{\tau}\right)^{-\alpha} + \frac{I(\tau_l/t; \lambda, 1 - \beta)}{\Gamma(\lambda + 1 - \beta)}\left(\frac{t}{\tau}\right)^{-\beta}\right]. \tag{22}$$

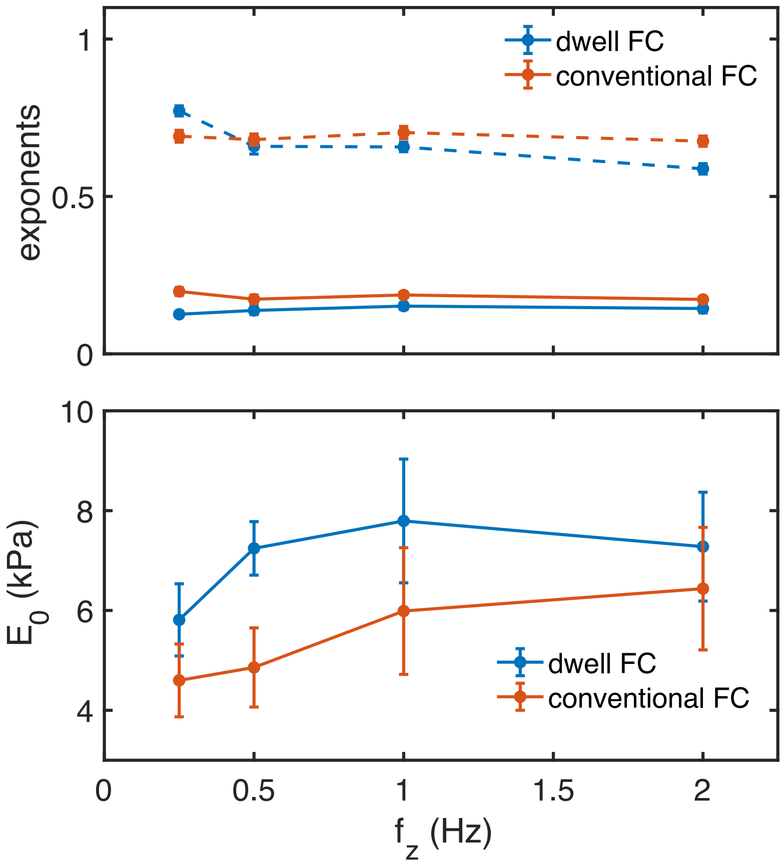

Figure S3: Dependence of the fitting parameters (E_0, α, β) on the vertical frequency f_z and type of force curve in AGP01 cells. This figure shows the effect of the loading speed (controlled by the vertical frequency f_z) and type of force curve in the fitted parameters for the AGP01 cells. The fitted data is nearly independent on f_z and on the curve type. Although dwell force curves are indicated for an accurate description of slow frequency response, both fast and slow frequency responses are present in the loading curve. If the loading curve is too fast (large f_z), then the cell may not have time enough to enter in the the slow relaxation regime, and the slow frequency response is only partially present in the loading curve. As rule of thumb, the exponent β obtained in conventional FCs is always equal or larger than the exponent β obtained with dwell FCs.

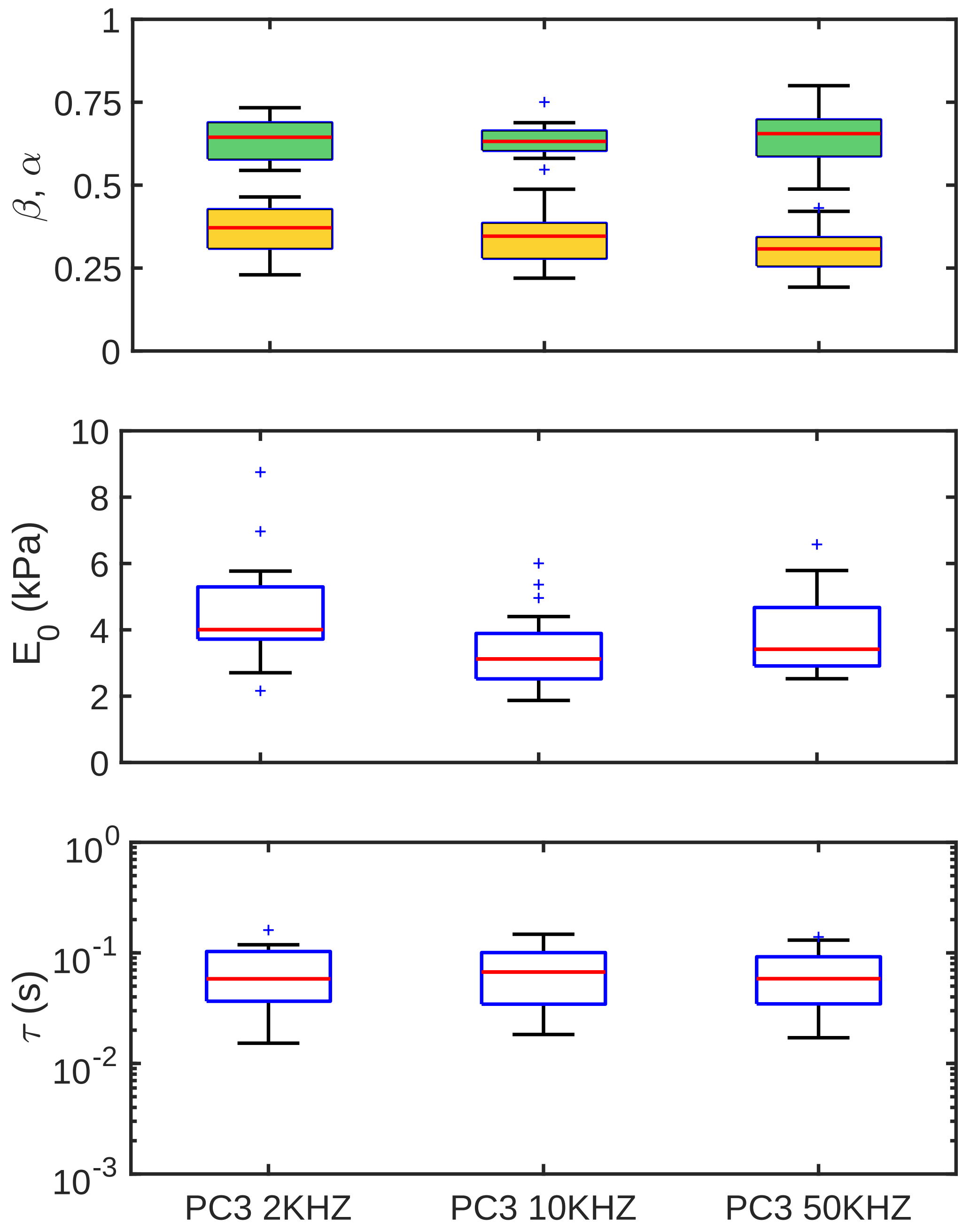

Figure S4: Comparison of results with different sampling frequencies for PC3 cells. The upper and lower whiskers represent the highest and lowest data within 1.0 IQR (interquartile range). Data outside this range is shown with a cross symbol. In the top panel, relaxation exponents α β are represented by green and yellow boxes, respectively. This figure shows the effect of different sampling frequencies on the fitted exponents in two types of cells. The individual data sets combines measurements performed with $f_z = 0.25$ Hz, 0.5 Hz, 1.0 Hz and 2 Hz. In the force curve mode, our AFM operates between 2 kHz and 50 kHz. For those frequencies, the shortest measurable time scales are 0.5 ms and 0.02 ms, respectively.

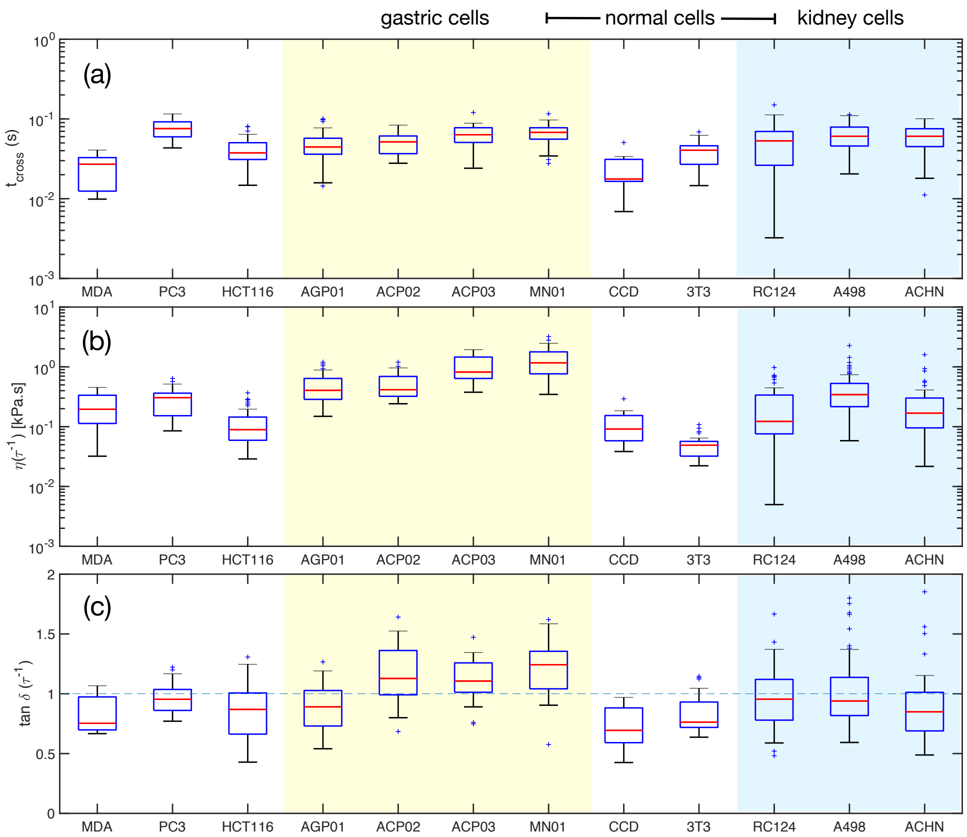

Figure S5: Box-and-whisker plots of the crossover timescale (a), effective viscosity (b) and phase angle (c) of all cells. In panel (c), $\tan\theta(\tau^{-1}) > 1$ ($\tan\theta(\tau^{-1}) < 1$) indicates cells with fluid-like (solid-like) behavior. The effective viscosity of the gastric cells at ω_{cross} between fast and slow relaxation regimes is also inversely proportional to the migration properties of the cells. Since the cytoplasm is a highly complex fluid, the whole cell crawling process induces internal movement of fluids, cytoskeleton fibers and organelles. It is then physically sound that an enhanced internal viscosity slows down the crawling process. It also clear that the reduced viscosity favors an enhancement of the invasiveness of ACP02 and AGP01 cells as compared to ACP03 and MN01. The connection between effective viscosity and migration are in qualitative agreement with previous works suggesting that cancer cells are less viscous than their normal counterparts (Ketene *et al.* Nanomedicine, 8:93, 2012). For the slow exponent, we observe that $\beta_{AGP01} < \beta_{ACP02} < \beta_{ACP03} < \beta_{MN01}$. However, a slightly different trend is observed for the fast exponent $\alpha_{AGP01} < \alpha_{ACP03} < \alpha_{MN01} < \alpha_{ACP02}$. Among gastric cells, the phase angle shows that AGP01 is the only cell with a predominant solid-like behavior ($\tan\theta(\tau^{-1}) < 1$), while the other cancer and normal cells all exhibit a predominant fluid-like behavior ($\tan\theta(\tau^{-1}) > 1$), as shown in the bottom panel. It is interesting to note that ACP02 exhibits the largest value of the fast exponent. We hypothesize that this enhanced fluid-like behavior of the fast cytoskeleton response compared to the other cells is somehow connected to the enhanced invasiveness of ACP02. One important aspect of the cells studied in this work is that they all exist in a viscoelastic state that is close to the solid-liquid transition, as shown by the phase angle (loss tangent) $\tan\theta(\omega) = G''(\omega)/G'(\omega)$ in the bottom panel. At the crossover, the fractional KV viscoelastic theory states that $\theta = (\alpha+\beta)\pi/4$. Thus, cells are predominantly solid (liquid) when $\alpha+\beta < 1$ ($\alpha+\beta > 1$). The different levels of fluidity of the cells certainly depends on their function and health state. Even within the same cell line, different cells may exhibit different degrees of fluidity.

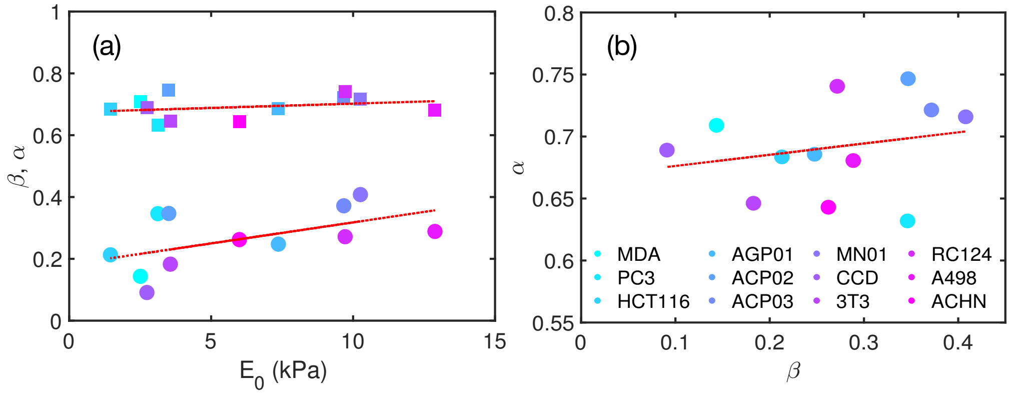

Figure S6: (a) Dependence of α and β with E_0. (b) Relationship between α and β. The red lines are guides for the eye. Panel (a) shows the correlation between α and β with E_0. Both exponents are proportional to E_0, but the dependence of β is much stronger. This suggests that the components responsible for the slow cell dynamics contributes much more to the overall cell stiffness than the components responsible for the fast dynamics. The weak dependence of E_0 with α shows that the cell cortex contributes very little to the cell stiffness, and this is compatible with the fact that the fast cytoskeleton response is caused by entropic fluctuations of the actin filaments. On the other hand, the strong dependence of E_0 with β shows that the whole cell stiffness is mainly due to the deformation and movement of the multi-component cytoplasmic crowd. Besides cell cortex, the f-actin represents a major component of the cytoplasm and its disruption increases the exponent β. The correlation between α and β in panel (b) also shows that they are weakly coupled as well. The four parameters, E_0, α, β, τ are not independent, and, in fact, this is not surprising. The degree of cytoskeleton crosslinking and the internal organization of the cytoplasmic crowd, roughly described by α and β, determines the overall cell stiffness. The cell cortex is anchored by many of the cytoplasmic filaments (actin, intermediate and microtubule filaments). Those filaments play an important role in the communication between different compartments of the cell. This qualitatively justifies the weak coupling between α and β. Quantitatively, the precise force propagation within cells remains unknown. These results are supported by the analysis of the Pearson's correlation coefficient given $\rho_{E_0,\alpha} = 0.28$, $\rho_{E_0,\beta} = 0.54$, $\rho_{\alpha,\beta} = 0.23$.

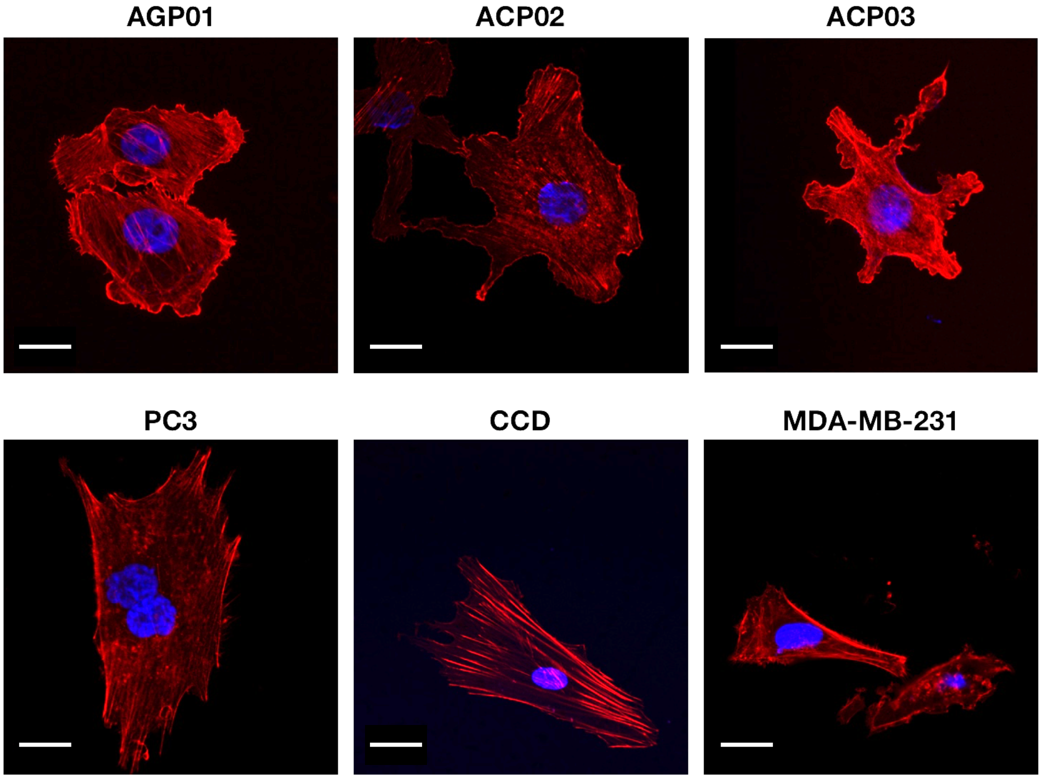

Figure S7: Confocal images of the f-actin network (red) and nuclei (blue) of the cells studied in this work, showing distinct cytoskeleton morphologies. The scale bar is of 20 μm.
